# Harnessing multi-omics approaches to decipher tumor evolution and improve diagnosis and therapy in lung cancer

**DOI:** 10.1186/s40364-025-00859-y

**Published:** 2025-11-05

**Authors:** Yicong Cheng, Ling Bai, Jiuwei Cui

**Affiliations:** Cancer Center, The First Hospital of Jilin University, 1 Xinmin Road, Changchun, 130021 P. R. China

**Keywords:** Multi-omics, Lung cancer, Tumor evolution, Tumor microenvironment, Cancer hallmarks, Cancer biomarkers

## Abstract

With the advancement of novel technologies such as whole-genome sequencing, single-cell sequencing, and spatial transcriptomics, single-omics analyses have already promoted the research of tumorigenesis as well as development and have partly elucidated the evolutionary processes of lung cancer. However, it is still difficult to distinguish these confounding features via single dimensional approaches due to the complexity, heterogeneity and cell-cell interactions with the immune microenvironment in lung cancer. Multi-omics approaches provide a holistic framework for constructing detailed tumor ecosystem landscapes, thereby facilitating the development of a more robust classification system for precision diagnosis and treatment, and aiding in the discovery of novel cancer biomarkers. In this review, we summarize the potential and applications of multi-omics approaches in characterizing intratumor heterogeneity and the tumor microenvironment throughout the course of lung cancer development. By further discussing the discovery and application of diagnostic and therapeutic biomarkers across precancerous lesions, early-stage lung cancer, tumor progression, metastasis, and therapy resistance, we outline the current challenges and future prospects of using multi-omics to identify reliable biomarkers. Moreover, we emphasize that integrative multi-omics models hold great promise for elucidating the complex interactions within the lung cancer ecosystem, thereby contributing to improved diagnostic accuracy, optimized therapeutic strategies, and better patient outcomes.

## Background

Lung cancer has become a significant global threat to human health, and it is estimated that lung cancer-related deaths will account for 20% of all cancer-related fatalities by 2024 [1]. Despite significant advancement in diagnostic strategies and effective treatments, the five-year survival rate for lung cancer remains below 20% in most countries [2, 3]. Therefore, it is crucial to enhance existing strategies for lung cancer diagnosis and treatment to achieve early detection, more precise therapeutic interventions, and comprehensive patient management.

**Fig. 1:**
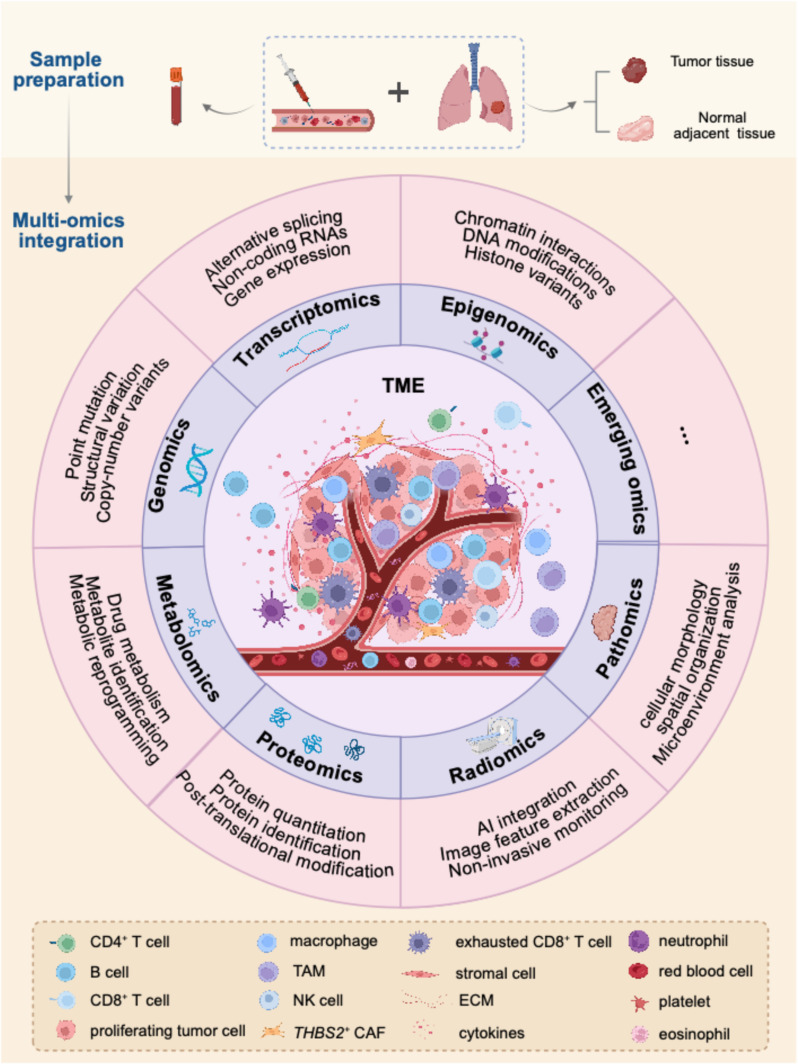
An overview of applying multi-omics approaches for lung cancer research. Multi-omics analysis is revolutionizing cancer research by offering a more holistic understanding of tumor ecosystem. Patient-derived samples, obtained from tumor and adjacent tissue as well as peripheral blood, can be analyzed by multi-omics approaches in multiple dimensions. These approaches encompass genomics, transcriptomics, epigenomics, proteomics, metabolomics, radiomics, pathomics and emerging fields. The integration of these multi-omics data sets, typically facilitated by machine learning techniques, enables the identification of complicated interactions within the TME. Multi-omics approaches provide significant insights into the mechanisms underlying tumor initiation, progression, and metastasis. Key applications of these methods include the identification of novel cancer biomarkers, mapping tumor evolution and the change in TME, addressing treatment resistance, and facilitating the development of personalized therapeutic strategies. The graphical abstract was created with BioRender.com

Single-omics approaches have contributed to various aspects of lung cancer research, such as identifying driver genes, characterizing the tumor microenvironment (TME), and capturing spatial tissue features. However, integrative multi-omics analysis provides a complementary, multidimensional view of tumor evolution, offering a more comprehensive understanding of intratumor heterogeneity (ITH) (Fig. 1).

Lung cancer constitutes a self-sustaining cellular ecosystem wherein cancer cells interact and collaborate with both cellular and non-cellular components within the TME, as well as distant organ niches [4, 5]. The emergence of multi-omics approaches has become vital in comprehensively understanding tumor characteristics and in the context of diagnosis, treatment along with prognosis. Multi-omics enables a more holistic investigation of cancer’s molecular mechanisms while facilitating biomarker discovery across multiple levels, offering the ability to construct dynamic tumor atlases across different dimensions [6]. Despite persistent challenges such as high cost, cross-platform variability, and data integration complexity—which necessitate advanced computational tools such as Seurat v5, Cell2location, Muon, iCluster, and multi-omics factor analysis—the accelerating development of integrative multi-omics is expected to transform both lung cancer research and clinical management.

To better capture the multifaceted complexity of lung cancer development, this review synthesizes current advances in the application of multi-omics approaches for identifying biomarkers across different stages of tumor evolution. It further highlights the strengths and limitations of individual omics platforms, the added value of integrative analyses, and the insights gained from linking diverse molecular layers. In addition, we summarize the clinical applications of multi-omics in lung cancer diagnosis and treatment, aiming to provide a more comprehensive framework for improving early detection, guiding personalized therapeutic strategies, and enhancing prognostic monitoring and evaluation.

## From single omics to multi-omics: scope and distinctive features (Table 1)

Single omics approaches such as genomics, transcriptomics, epigenomics, proteomics, metabolomics and radiomics have each substantially advanced our understanding of lung cancer biology and have contributed to biomarker discovery across the continuum of disease. Genomics, through whole-exome sequencing (WES) and whole-genome sequencing (WGS), has uncovered driver mutations such as epidermal growth factor receptor (EGFR), kirsten rat sarcoma viral oncogene (KRAS), tumor protein p53 (TP53) and primarily delineated evolutionary trajectories that underpin both early tumorigenesis and therapy resistance [7]. Transcriptomics has revealed differential gene expression patterns and pathway activation, while single-cell and spatial transcriptomics studies have identified rare subpopulations, such as exhausted T cells and transitional alveolar epithelial states, that are critical for prognosis and therapeutic response [8, 9]. Epigenomics has highlighted how aberrant DNA methylation, histone modifications, and chromatin remodeling regulate oncogene activation and tumor suppressor silencing, some of which have emerged as predictive biomarkers for immunotherapy [10]. Proteomics has bridged the gap between gene expression and functional output by mapping signaling networks, post-translational modifications, and druggable targets, while metabolomics has exposed rewired metabolic pathways—such as lactate accumulation and altered inositol metabolism—that drive immune suppression and therapy resistance [11, 12]. Radiomics has enabled the extraction of high-dimensional quantitative features from medical images, providing a powerful approach to quantify imaging phenotypes and uncovering ITH beyond visual interpretation [13].

**Table 1:** Overview of omics technologies and their representative features

	Technology type	Representative features	Problems addressed	Limitations	Example of application and future prospects
Single omics	Genomics	WES: sequence exons	Reveal genetic background, driver mutations, tumor evolutionary trajectories.	Limited ability to interpret non-coding regions and large structural variants	Uncover driver mutations such as EGFR, KRAS, etc; foundation of precision medicine; enable risk prediction and personalized therapy.
		WGS: sequence the entire genome		High cost, interpretation challenges, long processing time	
	Epigenomics	WGBS: DNA methylation sequencing	Reveal chromatin accessibility, transcription factor binding, and epigenetic regulation.	High cost, huge data requirement, and DNA degradation	Study drug resistance, cellular plasticity, and develop epigenetic therapies.
		ChIP-seq: histone modification profiling		Require high-quality antibodies and limited resolution	
		ATAC-seq: chromatin accessibility assays		Identify open chromatin regions, and high background noise in heterogeneous tissues	
		scATAC-seq: single cells		High cost, resolution limitations, and heavy computational demands	
	Transcriptomics	Bulk RNA-seq: differential expression and pathway activation	Identify differentially expressed genes, cellular heterogeneity, pathway activation.	Cell averaging, lacking accuracy in single-cell level, and no spatial context	Identify rare subpopulation such as exhausted T cells and transitional alveolar epithelial states, etc; combine with single-cell and multimodal sequencing to dissect microenvironment dynamics and immune states.
		scRNA-seq: identifies rare subpopulations		Limited information dimension, loss of spatial information, and difficulty in tracking dynamic evolution	
		Spatial transcriptomics: maps cell distribution and interactions within the TME		High cost, single molecular layer, dissociation between function and phenotype, and limited data integration and interpretation	
	Proteomics	Mass spectrometry (MS)	Bridge the gap between gene expression and function; reveal signaling networks and drug targets.	Limited sensitivity; low-abundance proteins are difficult to detect; quantification is challenging	Clinical biomarker discovery; drug target development; spatial proteomics is emerging.
		Antibody array			
	Metabolomics	NMR: non-destructive	Reveal metabolic pathway alterations, tumor–immune metabolic interactions.	Metabolites are easily influenced by sampling and environment; data interpretation is complex	Expose rewired metabolic pathways such as lactate accumulation and altered inositol metabolism. Integration with immuno-omics for immunometabolism research and drug response prediction.
		LC-MS/MS: polar/nonpolar metabolites			
		GC-MS: volatile and thermally stable metabolites			
	Radiomics	AI algorithms: extracts quantitative features from medical images (CT, PET, MRI)	Provide non-invasive biomarkers for early detection and diagnosis; assist in predicting prognosis and treatment response.	Lack of standardization in image acquisition, segmentation, and feature extraction; limited interpretability of AI-based models	Integration with multi-omics to uncover molecular characteristics; application in personalized therapy decision.
Multi-omics integration	Horizontal integration	Spatial transcriptomics + scRNA-seq	Uncover cellular heterogeneity, subpopulation structure, and spatial organization.	High cost and technical complexity, algorithm challenges	Discover KACs as the intermediate state in early-stage LUAD transformation; spatial maps in TME, tumor evolution studies, guiding precision immunotherapy.
	Vertical integration	Genomics: WES, WGS + Transcriptomics: RNA-seq, scRNA-seq	Enable precise multi-layered subtyping, link the genomic mutation and expression with specific cell subpopulations.	High cost, data integration, algorithm challenges	Uncover ITH and TME characteristics, guiding immunotherapy and target therapy.
		Genomics: WES, WGS + Transcriptomics: scRNA-seq + Metabolomics: LC-MS/MS	Provide multi-layered biomarkers, revealing cell subpopulation correlated with metabolic reprogramming.	High cost and sample requirement, specialized algorithms, further development in single cell metabolomics	Integration with spatial omics and deep learning machine, depicting the metabolic and immune landscapes in tumor evolution; construct genome-transcriptome-cellular network-metabolome model.
		Radiomics +Genomics: WES, WGS +Transcriptomics: RNA-seq +Metabolomics: LC-MS/MS, NMR	Reveal the connection between imaging phenotype, molecular mechanism and clinical outcome.	High cost, black-box interpretability issues, lack of standardization	Capture ITH and TME complexity more comprehensively in a non-invasive and dynamic way, guiding early diagnosis and prognostic stratification.

Collectively, these single-omics strategies have contributed to the identification of diagnostic biomarkers (e.g., mutation panels and high-dimensional radiomic features for early detection), prognostic biomarkers (e.g., transcriptomic and epigenetic signatures associated with survival), and predictive biomarkers (e.g., proteomic and metabolic correlates of immunotherapy response). However, their inherent limitations—restricted coverage (e.g., WES missing noncoding variants), loss of spatial and temporal resolution, and inability to capture cross-layer regulation—mean that no single approach can fully capture the complexity of lung cancer. These gaps highlight the need for integrative multi-omics frameworks that can connect genomic alterations with downstream transcriptional, epigenetic, proteomic, and metabolic consequences, thereby offering a more holistic and clinically relevant understanding of tumor evolution. The integration of multi-omics approaches typically use two major strategies: horizontal integration within the same omics layer and vertical integration across different biological layers.

Horizontal integration exemplifies how different technologies within the transcriptomic layer or omics in multiple dimensions can complement one another. For example, the combination of spatial transcriptomics and single-cell RNA sequencing (scRNA-seq) addresses the limitations of each approach when applied independently—such as the mixed-cell signals and resolution constraints of spatial transcriptomics, and the loss of spatial context in scRNA-seq. Together, these methods enable precise mapping of subcellular populations, revealing both their molecular states and their spatial organization within the TME . A notable example is the discovery of KRT8^+^ alveolar intermediate cells (KACs), located closer to tumor regions compared with alveolar cells, which represent an intermediate state in the transformation of alveolar type II (AT2) cells into tumor cells during early-stage lung adenocarcinoma (LUAD) [14]. By combining the high-resolution profiles of scRNA-seq with the spatial information from spatial transcriptomics, researchers can delineate cellular heterogeneity, spatial location, migratory behavior, and pathway activity at single-cell resolution, thereby offering deeper insights into tumor evolution. However, these analyses require larger sample sizes, result in higher costs, and demand rigorous algorithms to address batch effects and integration complexity. Additionally, radiomics can be integrated with multi-omics data (e.g., genomics, transcriptomics, and metabolomics) through joint analyses using machine learning or deep learning frameworks such as multimodal neural networks and iCluster. This multidimensional integration compensates for limitations of single omics alone, such as imaging phenotypes that lacks molecular interpretability in radiomics, lack of dynamic reflection in genomics, spatial heterogeneity in transcriptomics and metabolomics. The integration of radiomics and multi-omics provides non-invasive, whole-tumor assessment linked to molecular mechanisms, which may significantly improve the accuracy of early diagnosis, prognostic stratification, and therapeutic response prediction [15].

Vertical integration connects multiple biological layers, from genomics to transcriptomics to metabolomics, thereby linking genetic alterations to transcriptional dysregulation, metabolic reprogramming, and ultimately tumor–immune interactions. For example, WES and WGS identify driver mutations and structural variants at the genomic level. Bulk RNA-seq can then be used to verify whether these genomic alterations result in transcriptional dysregulation, highlighting pathways differentially expressed between tumor and normal tissues. scRNA-seq further refines this analysis by revealing which specific cell populations drive these transcriptional changes and by identifying subpopulations that carry particular mutations [16]. Projecting WES/WGS-derived mutation data onto scRNA-seq profiles allows the mapping of mutation-bearing cell types and the assessment of how these mutations reshape transcriptional states at the single-cell level [17]. Finally, metabolomics, through liquid chromatography-tandem mass spectrometry (LC-MS/MS) or related platforms, validates whether altered metabolic gene expression leads to measurable changes in metabolite levels and pathway activity [18]. This cross-layer workflow enables the construction of a genome (DNA)–transcriptome (RNA)–cellular network (scRNA-seq)–metabolome model, providing a multidimensional framework to explore lung cancer heterogeneity, the tumor–immune microenvironment, and therapeutic vulnerabilities.

Beyond these, other integrative strategies also contribute to biomarker discovery and therapeutic development. The combination of transcriptomics and proteomics bridges the gap between RNA expression and protein activity, improving the identification of functional biomarkers and druggable targets [19]. Similarly, integrating immunogenomics and microbiomics offers novel insights into how host immunity and the commensal microbiome jointly influence lung cancer progression and therapy response [20].

Collectively, these multi-omics strategies not only refine biomarker discovery of lung cancer but also reveal therapeutic vulnerabilities and resistance mechanisms with higher precision. By connecting diverse molecular layers, they establish a more clinically relevant framework for early detection, precision diagnosis, rational drug development, and improved prognostic evaluation in lung cancer. Despite persistent challenges such as high cost, cross-platform variability, and data integration complexity—which necessitate advanced computational tools such as Seurat v5, Cell2location, Muon, iCluster, and multi-omics factor analysis—the accelerating development of integrative multi-omics is expected to transform both lung cancer research and clinical management.

## Multi-omics analysis facilitates understanding of tumor molecular evolution

**Fig. 2:**
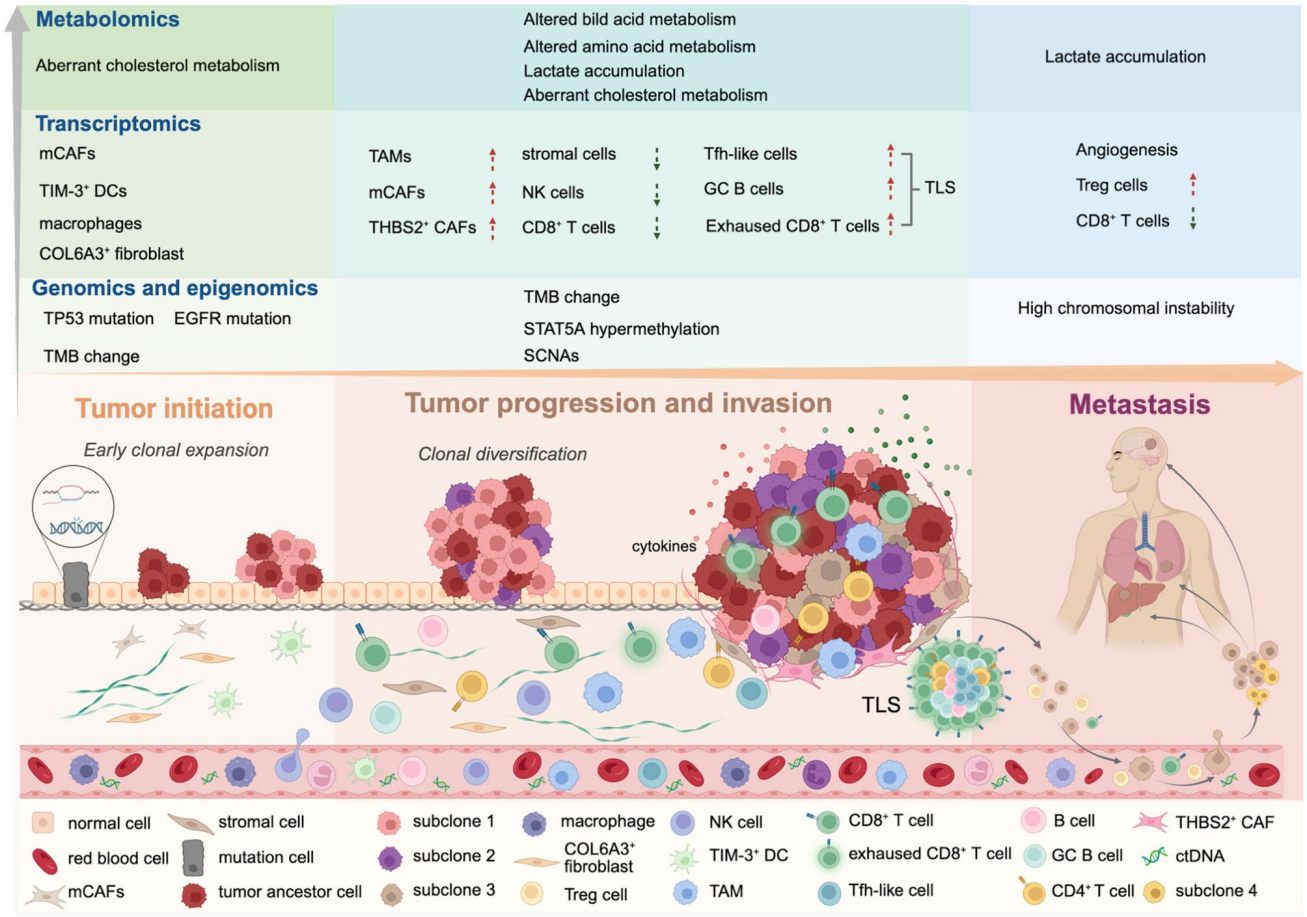
The integration of multi-omics approaches in the dynamic evolution of lung cancer. Multi-omics approaches not only help to map the clonal diversity and tumor evolution progress, but also identify biomarkers at each stage of progression. Tumor initiation is commonly driven by genetic mutations and early clonal expansion. High-throughput sequencing techniques enable the detection of point mutation such as TP53 and EGFR. Concurrently, mCAFs, TIM-3^+^ DCs, COL6A3^+^ fibroblasts are observed during the precancerous stage as well as alterations in cholesterol metabolism, setting the stage for clonal expansion. During tumor progression and invasion, genomics, transcriptomics and immunopeptidomics integrate in identifying the biomarkers of clonal diversification. Clonal diversification is characterized by copy number variations, including TMB and SCNA, as well as transcriptomic diversity, which contribute to ITH and subclonal variation. Simultaneously, immune infiltration and evasion are activated. The abundance and activity of THBS2^+^ CAFs, mCAFs, and TAMs increase. Furthermore, the composition of TLS shifts, with an increase in the abundance of Tfh-like cells, GC B cells, and exhausted CD8^+^ T cells. Metabolic alterations also occur during tumor progression, particularly during tumor invasion, where bile acid, cholesterol metabolism, glycolysis, and amino acid metabolism are reprogrammed. This results in enhanced cholesterol efflux, iron export, lactate accumulation and changes in key metabolites associated with amino acid metabolism, contributing to invasion in lung cancer. At the metastatic stage, dissemination of single subclone into the bloodstream, subsequent colonization and metastasis in other organs are reflected by the biomarkers via multi-omics approach. Energy metabolism is largely reprogrammed to favor aerobic glycolysis as evidenced by increased lactate production. Meanwhile, immune evasion biomarkers and angiogenesis promoters including VEGF and LRG1, are upregulated, contributing to the development of an immunosuppressive and nutrient-deprived TME. The graphical abstract was created with BioRender.com

Lung cancer can be conceptualized as a complex and dynamic ecosystem in which cancer cells interact not only with their clonal populations but also with the TME. The tumor ecosystem plays a pivotal role in shaping tumor evolution by exerting selective pressures that drive genetic and epigenetic alterations. Meanwhile, inflammation, immune infiltration and metabolic adaptations occur in the TME, facilitating tumor progression and metastatic to distant organ niches. The integration of multi-omics approaches provides a more comprehensive framework for tracking tumor evolution and identifying lung cancer biomarkers across multiple dimensions (Table 2). With a deeper understanding of tumor evolution, multi-omics analysis holds the potential to offer valuable insights into predicting progression, ultimately advancing precision medicine and enabling the development of personalized therapeutic strategies for lung cancer (Fig. 2).

**Table 2:** The application of multi-omics approaches in identifying tumor evolution mechanism of lung cancer

Stage	Multi-omics	Key results	Reference
Premalignant lesion and early-stage	scRNA-seq + Spatial transcriptomics	High TIM-3 expression is associated with reduced activity of antigen presentation pathways and impaired T-cell function, with these cells predominantly localized within precancerous lesions and adjacent tumor regions. TIM-3 is therefore identified as a potential biomarker for LUAD precancer interception.	[21]
	WES + RNA-seq + scRNA-seq + Spatial transcriptomics	During the progression from precancerous lesions (AAH and AIS) to early invasive lung adenocarcinoma (MIA), immune and non-immune cells undergo coordinated evolution, with angiogenesis playing a pivotal role in driving tissue invasion at the late AIS stage. In parallel, the study defines molecular subtypes associated with pathological and radiological patterns, providing potential strategies for early diagnosis and intervention.	[22]
	scRNA-seq + scATAC-seq	AT2 cells and their proliferating subsets are major contributors to lung cancer susceptibility.	[14]
	scRNA-seq + Spatial transcriptomics	KRT8^+^ AT2 cells can act as intermediate cell subsets in the transformation of AT2 in precancer to tumor cells in LUAD and drive KRAS mutations.	[23]
	scRNA-seq + Spatial transcriptomics + RNA-seq	An MPLC-specific CLDN2^+^ AT2 epithelial subtype that aggregates and signals with COL6A3^+^ fibroblasts is identified to distinguish MPLC from intrapulmonary metastasis.	[24]
	WES + Proteomics + Phosphoproteomics + Glycoproteomics	Aberrant cholesterol metabolism in AIS (precancerous stage) is a key event driving malignant transformation, identifies ER stress as a hallmark of progression to the invasive stage, and proposes PCSK9 and ER stress as potential targets for prevention and therapy.	[26]
Progression	WES + RNA-seq	Transcriptomic diversity via allele-specific expression and RNA editing is a major driver of ITH, improving metastasis potential combined with genomic features.	[27]
	RNA-seq + DIA + LC-MS	The overexpression of key glycolytic enzymes aldolase C and enolase 2 has been linked to increased lactate production and metabolic adaptation in LUAD.	[33]
	RNA-seq + scRNA-seq + RPPA + Spatial transcriptomics	THBS2^+^CAFs are identified as key biomarkers of progression, showing that high THBS2 levels correlate with poor survival, suppressed antitumor immunity, and poor response to immunotherapy.	[36]
	RNA-seq + DNA methylation sequencing + MS	Hypermethylation of STAT5A is associated with immune cell depletion in LSCC.	[37]
	WES + RNA-seq + scRNA-seq + DNA methylation sequencing + Spatial transcriptomics	LUAD progression and spatial heterogeneity are driven primarily by non-genetic, plastic transcriptional state shifts and TME reprogramming rather than new driver mutations.	[38]
	WES + RNA-seq + HLA-I/II MS immunopeptidomics + Spatial transcriptomics	CD8^+^ T-cell–excluded lung tumors have higher expression and HLA-I presentation efficiency of tumor-associated antigens with an enrichment of predicted neoantigens, while the HLA-II peptidome mirrors immune-cell composition.	[41]
	RNA-seq + scRNA-seq + RPPA	ECM-based “matreotypes” and a 28-gene matrix-risk signature are defined to predict progression of squamous carcinoma in situ, and ECM-High subtype is correlated with poorer prognosis as well as immuno-stromal remodeling.	[42]
	scRNA-seq + Spatial transcriptomics	The reprogramming of TAM facilitates cholesterol efflux from TAMs into the TME.	[43]
Metastasis	scRNA-seq + Spatial transcriptomics + WGS	Brain metastases are characterized by high chromosomal instability and enrichment of a neural-like CIN-high cancer-cell state alongside brain-adapted microenvironmental remodeling.	[46]
	Radiomics + Bulk RNA-seq	Low-risk NSCLC brain metastatic patients have the characteristics of upregulated interferon pathways, higher CD8⁺ T-cell infiltration, and pro-inflammatory ecosystems.	[47]
	WES + RNA-seq + scRNA-seq + DIA + RPPA + Targeted metabolomics	LC-BrMs retain most drivers yet show higher intratumor heterogeneity and focal amplifications, shift toward mitochondria-specific OXPHOS with immune suppression, and are vulnerable to OXPHOS inhibition that synergizes with anti-PD-1.	[48]
	RNA-seq + scRNA-seq + LC-MS/MS	LRG1 which can be upregulated in a STAT3–dependent manner by endothelial cells, has been identified as an indicator of metastatic progression.	[18]

### Multi-omics insights into premalignant lesions and early-stage lung cancer

During the precancerous and early stages of lung cancer, genetic and epigenetic alterations within cells, together with dynamic remodeling of cellular populations in the microenvironment, create an evolutionary context that favors early clonal expansion.

**Fig. 3:**
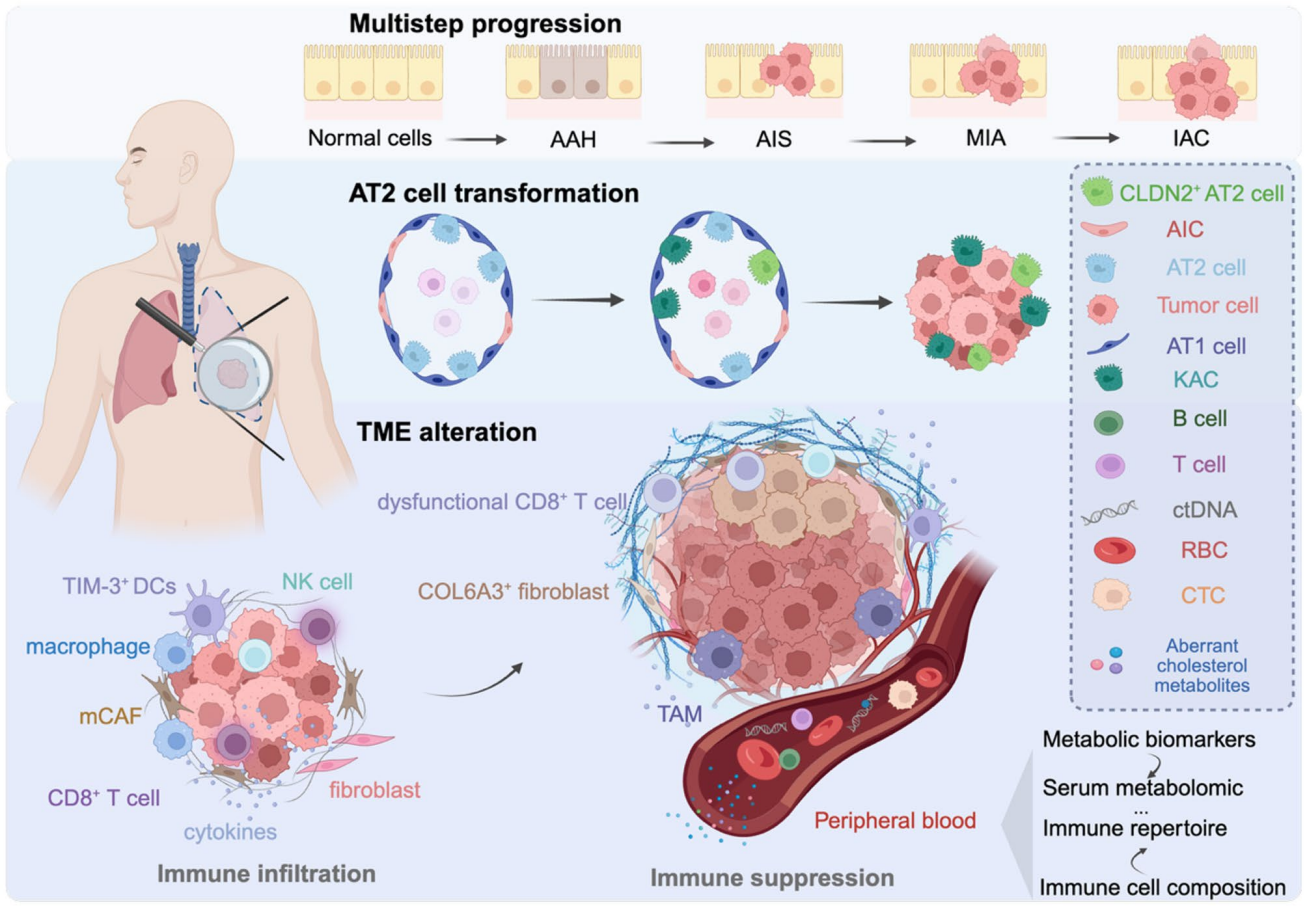
Characteristics of lung cancer at premalignant lesions and early-stage analyzed by multi-omics. The evolution of lung cancer is a dynamic progress that initiated from normal cells and progresses through AAH, AIS, MIA, and IAC. Through the integration of single-cell transcriptome, it has been revealed that contributing cell subsets such as AT2 cells may undergo proliferations and transformations into intermediate cells types like KACs via epigenetic modification. These transformations are pivotal in the formation of intratumor cells, which may mediate genetic susceptibility to lung cancer. In the TME, immune cells such as NK cells, macrophages, CD8^+^ T cells are recruited from peripheral blood, leading to immune infiltration. TIM-3^+^ DCs, COL6A3^+^ fibroblasts, and macrophages localize within precancerous lesions and adjacent area. The metabolic reprogramming, evidenced by metabolomic analyses, leads to aberrant cholesterol metabolism as the hallmark of AIS. These metabolic shifts may diminish immune infiltration and enhance immune suppression, such as the decrease and dysfunction of CD8^+^ T cell and NK cell, thereby promoting tumor growth and metastasis. Additionally, by employing serum metabolomics and immune repertoire analyses in peripheral blood, novel metabolic biomarkers and immune cell compositions can be further elucidated in the early stages of lung cancer. The graphical abstract was created with BioRender.com

Studies employing single-cell RNA sequencing (scRNA-seq) and spatial immune/transcriptomics profiling—by overcoming the limitations of spatial transcriptomics (mixed-cell signals) and scRNA-seq (loss of spatial information)—have revealed that high T-cell immunoglobulin and mucin-domain containing-3 (TIM-3) expression is associated with reduced activity of antigen presentation pathways and impaired T-cell function. These TIM-3^+^ cells are predominantly localized within precancerous lesions and adjacent tumor regions, thereby identifying TIM-3 as a potential biomarker for LUAD precancer interception [21]. Besides, the integration of WES with bulk RNA-seq has been used to discriminate pathological and radiological subtypes, revealing the contribution of EGFR/TP53 mutations and dynamic changes in tumor mutational burden (TMB) during early progression. Furthermore, joint analysis of spatial transcriptomics and scRNA-seq has delineated the spatial distribution and dynamic evolution of distinct cell types, uncovering the cooperative roles of angiogenesis and matrix-associated cancer-associated fibroblasts (mCAFs) in promoting early LUAD invasion [22]. To identify epithelial subpopulations that may drive or sustain tumorigenesis, integrated genomics and transcriptomics analyses have revealed that alveolar type II (AT2) cells and their proliferating subsets are major contributors to lung cancer susceptibility [14]. Combined scRNA-seq and spatial transcriptomics profiling further demonstrated that keratin 8–positive (KRT8^+^) alveolar intermediate cells (KAC) function as intermediate subpopulations in the transition from precancerous AT2 cells to malignant cells in early LUAD, and their presence is associated with poorer survival [23]. These KAC cells are enriched in non-malignant regions adjacent to tumors, exhibit increased plasticity, and are linked to KRAS-driven mutations. In addition, multi-omics analyses integrating scRNA-seq, RNA-seq and spatial transcriptomics have identified specific aggregation of claudin-2–positive AT2 (CLDN2^+^ AT2) cells and collagen type VI alpha 3 chain-positive (COL6A3^+^) fibroblasts as biomarkers to distinguish multiple primary lung cancers from intrapulmonary metastasis during the development of early-stage LUAD [24, 25]. Collectively, these findings refine the epithelial cell state atlas and elucidate the cellular and molecular mechanisms underlying early lung cancer development, providing potential therapeutic targets for preventing tumor initiation and progression (Fig. 3).

Beyond genomic change, early metabolic remodeling provides complementary biomarkers. An integrative framework spanning genomics, proteomics, phosphoproteomics, and glycoproteomics identifies aberrant cholesterol metabolism as a hallmark of adenocarcinoma in situ (AIS), potentially driving LUAD initiation [26]. Perturbation of proprotein convertase subtilisin/kexin type 9 (PCSK9) signaling decreases surfactant protein C and increases epithelial cell adhesion molecule expression, thereby promoting initiation, while sustained endoplasmic reticulum stress characterizes progression from AIS to invasive adenocarcinoma (IAC).

Overall, by situating immune, epithelial, and metabolic alterations within a single evolutionary continuum, multi-omics enables more accurate staging of precancerous lesions and supports earlier detection and rational early-intervention strategies.

### Multi-omics dissection of lung cancer progression

Lung cancer progression is a multistep evolutionary process shaped not only by genetic and transcriptional alterations within tumor cells but also by dynamic remodeling of the TME. Integrative multi-omics approaches have substantially advanced our ability to delineate both tumor-intrinsic evolution and TME remodeling, thereby providing a more comprehensive framework for identifying biomarkers and therapeutic vulnerabilities.

During the progression and metastasis of lung cancer, subclonal selection and clonal diversification frequently occur in many lung cancer driver genes and may exert stronger influence than ancestral clone selection in shaping tumor evolution [27]. ITH often manifests as spatial clonal diversity, characterized by branched evolutionary trajectories and shifts towards ancestral clones, which underlie tumor relapse [28]. By integrating WES and RNA-seq, it has been demonstrated that epigenetic dysregulation, allele-specific expression, and RNA editing are major contributors to ITH, interacting with DNA evolution to drive phenotype selection, progression, and metastasis in non-small cell lung cancer (NSCLC) [29]. A subset of highly and homogeneously expressed non-cancer genes exhibits stronger positive selection, while mutations in canonical cancer genes may undergo weaker negative selection [29]. In parallel, tumor progression is accompanied by metabolic reprogramming, which offers important opportunities for diagnostics and therapeutic intervention [30]. Metabolomic analyses have detected significant metabolic shifts during neoplastic transformation and invasion in LUAD, including alterations in bile acid metabolism, glutathione metabolism, β-alanine metabolism, nicotinamide/nicotinate metabolism, and arginine/proline metabolism [31, 32]. Integrative transcriptomics, proteomics, and metabolomics further identified that overexpression of glycolytic enzymes such as aldolase C and enolase 2 is linked to lactate accumulation and metabolic adaptation in LUAD [33]. These changes serve as biomarkers of enhanced biosynthetic capacity and adaptation to the TME.

The TME is a highly complex ecosystem composed of malignant and non-malignant cells spatially organized into distinct structural arrangements, playing a pivotal role in tumor initiation and progression [34]. Multi-omics has greatly improved TME characterization by identifying diverse cellular subtypes and interactions [35]. By integrating WES, bulk RNA-seq, scRNA-seq, and spatial transcriptomics, it was shown that the relative abundance of endothelial cells (ECs) and stromal cells decreases during the transition from normal lung tissue to atypical adenomatous hyperplasia (AAH) and early AIS. In contrast, tip ECs and mCAFs increase during later AIS, potentially promoting epithelial-mesenchymal transition, angiogenesis, and tissue invasion in LUAD [22]. Importantly, the abundance of immune cells, such as natural killer (NK) cells and CD8^+^ T cells, co-varies with ECs, highlighting links between vasculature and immune infiltration [22]. In addition, elevated thrombospondin-2-positive cancer-associated fibroblasts (THBS2^+^ CAFs) which are identified as biomarkers of progression, have been associated with reduced immune infiltration and poor response to immunotherapy in early-stage LUAD by integrating RNA-seq, scRNA-seq, proteomics, and spatial transcriptomics [36]. Genetic alterations and compositional shifts in TME components can establish an immunosuppressive microenvironment. For example, integrative analyses of RNA-seq, DNA methylation, and proteomics reveal that STAT5A hypermethylation is associated with immune cell depletion in lung squamous cell carcinomas (LSCC), enriched in immune-cool tumors and accompanied by decreased dendritic cell and effector signatures [37]. Multi-omics and spatial profiling also demonstrate that LUAD histologic progression is driven by epigenetic and transcriptional reprogramming, remodeling both tumor-intrinsic states and TME. Tavernari et al. showed that transition from lepidic to solid patterns involves distinct TME remodeling by integrating WES, RNA-seq, scRNA-seq, DNA methylation, and spatial transcriptomics [38]. Lepidic regions exhibite features of normal lung and an “immune desert” phenotype, whereas acinar regions show high immune infiltration, often enriched with B cells and tertiary lymphoid structures (TLS) resembling germinal centers (GC). TLS formation in early stages begins with CD4^+^ T-cell and B-cell aggregation around precancerous epithelium, followed by NK cell reduction. As tumors advance, TLSs evolve with enrichment of T follicular helper cell (Tfh)-like cells, GC B cells, and dysfunctional CD8^+^ T cells [39, 40]. By contrast, solid regions display an “immune-excluded” phenotype: immune cells accumulate at the periphery but are absent in the core, which expresses proliferative (Ki-67, Pan-AKT) and immunosuppressive markers (IL7R). Residual infiltrates are enriched in Treg markers (FOXP3, CD25, TIM-3), suggesting a highly suppressive niche. Further, integrative analysis of immunopeptidomics, genomics, transcriptomics, and spatial profiling revealed that LUAD progression is shaped by distinct immune surveillance modes. Tumors segregate into inflamed, T cell–infiltrated versus immune-excluded phenotypes: the former enriched for human leukocyte antigen class II (HLA-II) presentation and immune editing, the latter preserving self-antigen presentation and latent neoantigen potential [41]. Additionally, extracellular matrix (ECM) remodeling has been identified as a key driver of NSCLC initiation and progression. By integrating bulk RNA-seq, scRNA-seq, WES, and proteomics, researchers delineated ECM-driven stromal–immune states that shape lung squamous cell carcinoma (LUSC). A 28-gene matrix risk signature shows that ECM remodeling emerges at pre-invasive stages and stratifies tumors into prognostic “matreotypes”. The ECM-high matreotype is characterized by enrichment of tumor-associated myofibroblasts, depletion of AT2 and EC compartments, higher Treg/Tfh/B-cell signals suggestive of TLS, together with angiogenesis and inflammatory activation [42]. Moreover, multi-omics profiling of tumor-associated macrophages (TAMs) via scRNA-seq and spatial transcriptomics revealed that altered cholesterol metabolism contributes to tumor-supportive TME. Overexpression of the cholesterol exporter ATP-binding cassette sub-family A member 1 (ABCA1) and downregulation of low-density lipoprotein receptor (LDLR) in TAMs promote cholesterol efflux into the TME, creating a lipid-rich environment that facilitates tumor survival and progression [43].

In summary, multi-omics dissection of lung cancer progression demonstrates that tumor-intrinsic alterations, including clonal diversification, epigenetic dysregulation, and metabolic reprogramming, act in concert with microenvironmental remodeling, including angiogenesis, immune suppression, ECM remodeling, and TAM-driven lipid metabolism, to shape disease evolution. These integrative insights not only deepen our understanding of LUAD biology but also provide crucial biomarkers for assessing aggressiveness, immune evasion, and therapeutic targeting.

### Multi-omics dissection of lung cancer metastasis

Metastasis consistently originates from the expansion of subclones. Ancestral clones may be selectively suppressed by chemotherapy, leading to the emergence of subclones derived from the relapse-initiating ancestral clone [44]. This process has been elucidated through the integration of WES, WGS and transcriptome sequencing. During the metastasis progression, important factors to infer metastatic potential corelated to the evolutionary context of the mutations within the tumor region are a lower proportion of subclonal mutations that are not regionally dominant and a decreased phylogenetic trunk [29]. Notably, findings from TRACERx indicate that most metastases diverge following the last clonal sweep within the primary tumor, with ancestral clonal mutations predominantly persisting in metastatic lesions [45]. Additionally, using scRNA-seq, spatial transcriptomics, and WGS in NSCLC, study shows that brain metastases are marked by high chromosomal instability and a recurrent chromosomal instability high (CIN^high^) neural-like cancer cell state that coexists in primaries and becomes enriched in the brain. Brain metastases also display a brain-adapted, immunosuppressive TME, with reduced CD8^+^ T cell infiltration, increased Tregs, diverse myeloid populations and perivascular signaling that promote angiogenesis and immune evasion [46]. By integrating radiomics and bulk RNA-seq, the ITH characteristics of low-risk NSCLC brain metastatic patients are shown as upregulated interferon pathways, higher CD8⁺ T-cell infiltration, and pro-inflammatory ecosystems [47].

Moreover, metabolic reprogramming and angiogenesis play critical roles in tumor metastasis by fostering a proliferative microenvironment conductive to tumor cell survival while concurrently driving immune evasion. For instance, integrating genomics, transcriptomics, proteomics, metabolomics and scRNA-seq on paired primary lung tumors and brain metastases, the study illustrates that lung cancer brain metastasis (LC-BrM) displays higher intratumor heterogeneity with LC-BrM-enriched focal somatic copy number alterations (SCNAs). And patients that tend to have an oxidative phosphorylation (OXPHOS)-high, immune-cold state that is vulnerable to the mitochondrial inhibitor gamitrinib predicts poor survival [48]. Primary tumors systemically reprogram vascular endothelium to perturb homeostasis and to precondition the vascular niche for metastatic growth. Leucine-rich alpha-2-glycoprotein 1 (LRG1) is an endothelial cell-associated vascular biomarker which can be upregulated in a STAT3–dependent manner by endothelial cells. LRG1 has been identified as an indicator of metastatic progression by integrating RNA-seq, scRNA-seq and serum proteomics [18].

## Utilization of multi-omics analysis in the diagnosis of lung cancer

Liquid biopsy approaches such as circulating tumor DNA (ctDNA), cell-free DNA (cfRNA), circulating tumor cells, and exosome detection have gained considerable attention in recent years due to their advantages of being non-invasive, repeatedly accessible, and enabling real-time monitoring [49–51]. However, their current applications remain limited by insufficient sensitivity and inadequate capture of dynamic changes, resulting in restricted diagnostic accuracy and reduced utility in lung cancer diagnosis. By contrast, integrative multi-omics strategies provide in-depth analysis across multiple molecular layers, which can compensate for the shortcomings of liquid biopsy, enhance early detection accuracy, and reveal tumor heterogeneity and microenvironmental remodeling mechanisms, thereby holding greater promise for advancing lung cancer diagnosis and precision therapy [52, 53].

### Current classification of the molecular subtypes in SCLC and NSCLC

Small cell lung cancer (SCLC) is genetically characterized by the presence of TP53 mutations and the loss of retinoblastoma 1 (RB1) in majority. Neuroendocrine (NE) subtypes of SCLC are classified based on the distinct expression of key transcription factors, including achaete-scute family bHLH transcription factor 1 (ASCL1) and neuronal differentiation 1 (NEUROD1) [54]. In addition to these well-established NE biomarkers, emerging non-NE SCLC biomarkers have been identified, including YES1-associated protein and an inflamed phenotype characterized by the high expression of genes related to interferon-γ (IFNγ) activation and immune checkpoints [55]. Notably, SCLC phenotypes exhibit dynamic plasticity throughout disease progression, highlighting the necessity of continuous monitoring to capture the evolving complexity of tumor subtypes, clonal evolution, and resistance mechanism.

NSCLCs comprise a heterogeneous group of malignancies classified by histopathological characteristics, including LUAD, LSCC, large cell carcinoma, and mixed-type carcinomas [56]. Advances in diagnostic technologies have facilitated the identification of molecular signatures that precisely distinguish NSCLC subgroups driven by distinct oncogenic alterations. Notably, mutations in EGFR and KRAS are among the prevalent molecular drivers of NSCLC. Despite these advancements, several limitations persist, necessitating further refinement of diagnostic approaches to improve accuracy and comprehensiveness. Conventional detection methods often fail to capture the full complexity of early-stage NSCLC, particularly in the context of tumor heterogeneity and evolving molecular landscapes. By integrating multi-layered data, multi-omics approaches address these challenges, offering a more comprehensive and precise characterization of NSCLC subtypes. This integrative strategy enhances early detection, facilitates personalized treatment strategies, and ultimately improves clinical outcomes.

### Emerging biomarkers in the diagnosis of SCLC and NSCLC

#### Integration of multi-omics approaches promotes classification of lung cancer

Multi-omics approaches help to accurately classify the phenotypes of SCLC and NSCLC, which is essential for guiding optimal therapeutic decision-making [57] (Table 3). Multi-omics analysis enables the classification of SCLC into four distinct molecular subtypes (NMF1-4), thus facilitating more precise and targeted therapeutic strategies by integrating WES, RNA-seq, proteomics and phosphoproteomics [58]. Each SCLC subtypes exhibits unique molecular characteristics: NMF1 is characterized by high cell cycle and DNA-damage signatures. This subtype has high proliferative activity and neuroendocrine features, which shows strong replication stress and are vulnerable to DNA-damage targeting therapies. NMF2 is defined by the highest TMB and significantly elevated expression of inhibitory Notch ligand delta-like protein 3 (DLL3). NMF3 subtype is distinguished by the activation of receptor tyrosine kinases (RTKs) signaling and extracellular-matrix remodeling signatures, while NMF4 subtype is marked by high MYC expression and the enrichment of MYC-related pathways. In addition to transcriptomic and proteomic profiling, tumor tissue and cfDNA methylation profiles have also been implemented for subtype classification, offering further insight into ITH [59].

The integration of multi-omics approaches has significantly advanced the classification and diagnostic methodologies in NSCLC. A proteogenomic analysis has identified five distinct multi-omics subtypes of NSCLC, each defined by unique molecular characteristics [60]. Subtype 1 (Metabolic) represents predominantly EGFR- and TP53-mutant LUAD (often female) with frequent chromosomal instability and cyclin dependent kinase inhibitor 2 A (CDKN2A) loss, marked by upregulated OXPHOS and other metabolic pathways. Subtype 2 (Alveolar-like) is largely EGFR mutant, genomically stable LUADs with low TP53 mutation/TMB burden, and also enriches for interleukin-33 (IL-33) and Notch pathway phosphorylation. It is consistent with an early-stage, oncogene-driven alveolar differentiation phenotype. Subtype 3 (Proliferative) is mostly male smoker patients with LSCC which is a chromosomally unstable, tumor-suppressor-deficient proliferative subtype, featuring TP53 mutations as well as strong upregulation of cell-cycle pathways. Subtype 4 (Hypoxic/Mesenchymal) occurs with high metastasis frequency which shows hypoxia pathway activation and neutrophil-degranulation signaling. It is chromosomally stable with a mesenchymal phenotype and is associated with the worst prognosis. Subtype 5 (Immunogenic), a KRAS-mutant and immune-hot subtype, is characterized by high tumor-infiltrating lymphocytes and has relatively favorable prognosis. This subtype owns the best survival among subtypes especially with adjuvant therapy. Additionally, radiomics combined with genomics and transcriptomics reveal the relationship between imaging manifestations and the immune status of the TME, which will help the classification and predict immunotherapy responses in lung cancer. Through radiomics, target DNA sequencing, and bulk RNA-seq, radiomics-defined clusters recapitulate LUAD progression—indolent/differentiated (Cluster 1), developmental but low-proliferative (Cluster 4), and highly proliferative (Cluster 2–Cluster 3)—separated immune-activated (Cluster 2) from immune-suppressed (Cluster 3) tumor microenvironments in solid nodules, and yield a radiomics-related RNA signature that stratifies early LUAD prognosis [15].

#### Integration of multi-omics approaches enhances early detection of lung cancer

Beyond molecular classification, multi-modal platforms have significantly enhanced the early detection and diagnosis of NSCLC as more comprehensive tools. (Table 4) Among these, DNA methylation-based biomarkers, identified through multi-omics analyses have purported significant potential for diagnosis impact in NSCLC [61]. For instance, the PulmoSeek Plus Model, a multi-omics model that incorporates clinical features, radiomic data, and cfDNA methylation biomarkers, has been developed to optimize diagnostic accuracy and facilitate the non-invasive risk stratification of indeterminate pulmonary nodules [62]. This multi-modal demonstrates a robust diagnostic performance, with an area under the curve (AUC) of 0.90, a sensitivity of 0.88, and the specificity of 0.98 in combined set. Another multi-omics model based on genomic features of ctDNA, epigenetic signatures and other mutations information has been developed for early-stage NSCLC screening, which shows high specificity (96.3%) and reaches an AUC of 0.912 in the validation set enrolled from multiple centers, by integrating WGS-based cfDNA fragmentomics, genomics, and cfDNA methylomics [63]. The multiplex digital methylation-specific PCR (mdMSP) platform, a multiplex platform with integration of cfDNA methylation profiling and multiplex digital MSP, has been developed to distinguish NSCLC patients based on DNA methylation-based biomarkers (SOX17, CDO1, TAC1 and HOXA7) with high sensitivity and specificity [64]. This multi-modal approach achieves high sensitivity (90%) and specificity (82%), with a corresponding AUC of 0.86 in clinical performance.

**Table 3:** The application of multi-omics approaches in the classification of lung cancer

Lung cancer type	Multi-omics approaches	Subtype	Clinical implication	Reference
SCLC	WES + RNA-seq + TMT-based proteomics + Phosphoproteomics	SCLC nmf1: High proliferation with strong cell cycle and DNA-damage	Guide biological diagnosis and treatment selection.	[58]
		SCLC nmf2: Highest TMB; high DLL3 protein expression		
		SCLC nmf3: Increasing activity of RTKs signaling and extracellular-matrix remodeling		
		SCLC nmf4: High expression of MYC target and altered RNA metabolism pathway		
NSCLC	WES + RNA-seq + TMT-based proteomics + Phosphoproteomics + Acetylproteomics + scRNA-seq	Subtype 1: EGFR/TP53-mutant LUAD; female-enriched; metabolic pathway upregulation	Predict early-stage cancer and benefit from ICI.	[60]
		Subtype 2: EGFR-mutant LUAD; phosphorylation in IL-33 and Notch pathway; early-stage alveolar differentiation tumor		
		Subtype 3: TP53-mutant LSCC; male smoker-enriched; tumor-suppressor-deficient phenotype		
		Subtype 4: Poor prognosis; high metastasis frequency		
		Subtype 5: KRAS mutation; immune-hot tumor; better prognosis; better responsiveness to adjuvant therapy		
LUAD	Radiomics + NGS + RNA-seq	Cluster 1: Indolent/differentiated; best prognosis	Predict early LUAD prognosis.	[15]
		Cluster 2: High proliferation, immune-activated solid nodules		
		Cluster 3: High proliferation, immune-suppressive solid nodules with invasion		
		Cluster 4: Developmental but low-proliferative		

**Table 4:** The application of multi-omics approaches in the early detection of lung cancer

Model	Multimodal integration	Biomarkers	Clinical implication	Reference
PulmoSeek Plus Model	cfDNA methylomics + Radiomics	Clinical features, radiomic data, cfDNA methylation biomarkers	Classify pulmonary nodules accurately and facilitate early-stage diagnosis of NSCLC.	[62]
A modified WGS-based high-performance infrastructure for multi-omics method	WGSs-based cfDNA fragmentomics + Genomics + cfDNA methylomics	3 genomic features, co-methylation features and ctDNA mutations	Enhance the diagnostic performance of early-stage lung cancer and minimal residual disease.	[63]
mdMSP	cfDNA methylation profiling + mdMSP	DNA methylation-based diagnostic biomarkers (SOX17, TAC1, CDO1 and HOXA7)	Use in conjunction with LDCT for improving screening accuracy of early-stage NSCLC in high-risk population.	[64]
Clinic-RadmC Model	cfDNA fragmentomics + Epigenomics + Radiomics	Clinical data, fragmentomic profiles, and radiomic features through deep learning techniques	Optimize the diagnostic accuracy for indeterminate pulmonary nodules.	[66]
Deep learning-based multimodal platforms	NPLDI-MS + Radiomics	Serum metabolic fingerprint	Early detection of LUAD and pulmonary nodule management.	[67]
Electronic nose with volatolomics	Breathomics	Exhaled breath biomarkers	E-nose could be used alongside imaging as part of a future multi-omics platform.	[69]
Microbiome-based classifier	Microbiomics	Taxonomic features and airway microbiome shifts	Further combine with other omics forming multi-omics model.	[70]

Recent advancements in artificial intelligence (AI) and multimodal integration have significantly contribute to enhancing early diagnosis in lung cancer. Radiomics, which can be used in AI paradigm, combines with other multi-omics approach to provide non-invasive detective tools and to reveal the connection between imaging phenotypes, molecular mechanisms, and clinical outcomes through multidimensional information [65]. The integration of radiomics and epigenomics features (DNA methylation, histone modifications, and chromatin accessibility) can capture subtle differences in pulmonary nodules and detects molecular changes in early-stage lung cancer, enhancing the sensitivity and specificity for early-stage detection. For instance, Clinic-RadmC, a multi-omics model, combines clinical data, fragmentomics profiles, epigenomics and radiomics features through deep learning techniques to optimize the diagnostic accuracy for indeterminate pulmonary nodules [66]. This non-invasive strategy ahieves an AUC of 0.923, surpassing many single-omics models. Furthermore, radiomics combined with metabolomics correlate metabolic reprogramming via liquid chromatograph-tandem mass spectrometry (LC-MS/MS) with imaging findings and discover non-invasive detective biomarkers of the metabolic-immune microenvironment. Deep learning-based multi-omics platforms that integrate serum metabolic fingerprints with conventional clinical indicators, such as carcinoembryonic antigen (CEA) and image features via nanoparticle-assisted laser desorption/ionization mass spectrometry (NPLDI-MS) and radiomics, have demonstrated an AUC of 0.778 and a sensitivity of 81.79%, highlighting the add value of the combination of AI and multi-omics cancer biomarkers in developing multimodal platforms [67]. The integration of these multiplexed assays with AI-driven analytical strategies holds great potential for enhancing early LUAD diagnosis and refining pulmonary nodule classification.

Exhaled breath and volatolomics analysis have also emerged as promising non-invasive methods for the early detection of lung cancer [68]. Exhaled breath biomarkers, including cells, DNA fragments, and volatile organic compounds, can be analyzed using advanced techniques such as mass spectrometry, gas chromatography, and colorimetric sensors. For example, electronic nose has demonstrated high diagnostic performs in early-stage lung cancer detection, which yields high diagnostic rate of 86% and high F1 score of 92.5%. Its diagnostic accuracy aligns well with histopathological findings in clinical stage I lung cancer, suggesting its potential role in forming multi-omics model alongside imaging or other omics methods [69]. Moreover, bronchial microbiome profiling has facilitated the development of a microbial-based classifier for risk prediction and early detection of NSCLC [70]. This approach leverages distinct microbial signatures associated with lung cancer pathogenesis, further expanding the scope of multi-omics-driven diagnostics.

## Multi-omics analysis facilitates the implementation of biomarkers for lung cancer precision treatment

Gene mutations such as TP53, KRAS, and BRAF mutations and programmed death-ligand 1 (PD-L1) protein expression have shown promising results in predicting response to immunotherapy in lung cancer [71, 72]. However, PD-L1 expression is heterogeneous in spatially as well as temporally and can vary across different tumor stages and metastatic sites, making it a less reliable standalone biomarker [73]. Additionally, single-biomarker evaluations often exhibit lower sensitivity and fail to provide a comprehensive prognostic assessment compared to multi-omics approaches. Lung cancer biomarkers discovery, when integrated through multimodal methods, will offer a more robust strategy for tracking prognosis, understanding drug resistance mechanisms, and optimizing immunotherapy response predictions in lung cancer (Table 5).

**Table 5:** The utilization of multi-omics approaches in the understanding of drug-resistance and assessing the prognosis of lung cancer

Multi-omics approaches	Key results	Clinical implication	Reference
scRNA-seq + Spatial transcriptomics	COL11A1^+^ CAFs and SPP1^+^ macrophages are identified as key contributors to immune evasion in NSCLC, showing that these cells at the tumor boundary inhibit T-cell infiltration.	The study suggests COL11A1^+^ CAF as a potential biomarker to predict patient response to immuno-chemotherapy.	[74]
RNA-seq + LC-MS/MS	ACh metabolism via M3R and WNT signaling, contributes to drug tolerance and tumor relapse in EGFR-mutant NSCLC.	Pharmacological inhibition of ACh/M3R signaling via darifenacin shows promise in delaying tumor relapse and enhancing the response to EGFR-TKI treatment in NSCLC, suggesting a new therapeutic strategy to overcome drug resistance.	[75]
RNA-seq + WES + scRNA-seq	Acquired resistance to PD-1 blockade in NSCLC is characterized by elevated IFNγ signaling, immune dysfunction, and mutations in antigen presentation, which contributes to immune evasion.	The alterations in IFNγ and persistently-inflamed TME may provide new therapeutic biomarkers for reversing acquired resistance to PD-1 blockade treatment in NSCLC.	[76]
WES + RNA-seq	BTG2 methylation and expression serve as reliable prognostic biomarkers for early-stage NSCLC, with higher BTG2 expression associated with better survival outcomes.	This prognostic model may have new applications for future adjuvant trials and improve survival prediction.	[78]
scRNA-seq + Olink proteomics	Pretreatment levels of SP-B-specific immunoglobulin G autoantibodies and CD4^+^ T cells are associated with the development of developing ICI-induced irAEs.	These antibodies may serve as predictive biomarkers for an increased risk of developing ICI-induced irAEs.	[79]
scRNA-seq + DSP	The study reveals ITH in SCLC, with distinct immune profiles between NE-high (SCLC-A, SCLC-N) and NE-low (SCLC-P) subtypes, showing that REST expression correlates with immune infiltration and prolonged survival.	REST expression could be used as a biomarker to identify SCLC patients who may benefit from immunotherapy, potentially improving patient stratification and therapeutic outcomes.	[80]
WES + RNA-seq + scRNA-seq + DNA methylation profiling	PRAME is identified as a critical gene associated with LUAD recurrence, with its hypomethylation leading to overexpression. AT2 cells, immune exhaustion, and altered macrophage populations are key features of the TME contributing to recurrence.	The study identifies PRAME and TME features as potential therapeutic targets of stage I NSCLC recurrence for recurrence risk stratification and precise treatment.	[81]
Radiomics + Pathomics + NGS	A multimodal framework that fuses CT radiomics, PD-L1 immunohistochemistry and genomics predicts PD-L1 response in advance NSCLC (AUC = 0.80) and outperforms standard single biomarkers.	The model enables earlier risk stratification and treatment selection from routine clinical data.	[83]
Serum LC–MS + scRNA-seq	A multi-omics framework integrating pinpoints pre-metastatic, organ-specific biomarkers (e.g., SAA1 for bone) and maps intermediate cellular states.	The framework enables accurate prediction of metastatic trajectories at single-cell resolution proposes, assisting earlier detection and site-directed surveillance of LUAD metastasis.	[85]

### Multi-omics analysis facilitates understanding drug resistance of lung cancer

Multi-omics strategies play a vital role in identifying lung cancer biomarkers associated with drug resistance. The TME associated with resistance to immunotherapy is characterized by an insufficient antitumor immune response, highlighting the potential of immune-related cells as predictive biomarkers of drug resistance. For example, secreted phosphoprotein 1-positive (SPP1^+^) macrophages, collagen type XI α1 chain-positive cancer-associated fibroblasts (COL11A1^+^ CAFs) and inactivated TLSs, may serve as biomarkers associated with poor prognosis in the TME before and after immune checkpoint blockade (ICB)-chemotherapy in NSCLC via scRNA-seq and spatial transcriptomics analysis [74]. SPP1^+^ macrophages and tumor cells colocalize with COL11A1^+^ CAFs to stimulate the deposition and entanglement of collagen fibers at tumor boundaries, obstructing T cell infiltration and leading to poor prognosis in NSCLC. TLSs are divided into four stages: early lymphoid-aggregate TLSs (high expression of LTi markers and low cytokine expression), activated TLSs (high cytokine expression), declining TLSs (decreased cytokine expression), late TLSs (extremely low cytokine expression). And the independent cohorts show that activated TLS is associated with better overall survival (OS). Furthermore, specific signaling pathways and proteins can be biomarkers associated with drug resistance. Acetylcholine (ACh) metabolism has emerged as biomarkers involved in drug tolerance to EGFR tyrosine kinase inhibitors (TKI) therapy in the targeted therapy of NSCLC. ACh is specifically accumulated in drug-tolerant persister cells, leading to drug tolerance in EGFR-TKI therapy in part via WNT signaling through Ach muscarinic receptor 3 (M3R) by integrating RNA-seq and LC-MS/MS [75]. And darifenacin, an FDA-approved M3R inhibitor, is discovered to reduce tumor relapse significantly in vivo. Another multi-omics analysis has implicated IFNγ in acquired resistance towards PD-L1 blockade in NSCLC. WES, RNA-seq and scRNA-seq analysis illustrated that acquired resistance towards PD-L1 blockade is associated with elevated gene expression related to IFNγ, OXPHOS and DNA repair pathway response as well as chronic inflammation status in TME, further underscoring the complex mechanisms of immunotherapy sensitivity and resistance [76].

### Multi-omics analysis facilitates discovering prognostic biomarkers for lung cancer

Multi-omics analysis has shown promise in the discovery of novel biomarkers which help to predict clinical outcomes in lung cancer. In early-stage NSCLC, recent therapeutic breakthroughs have demonstrated improvements in prolonging the OS [77]. A multi-omics study integrating WES and RNA-seq has identified B-cell translocation gene 2 (BTG2), a tumor suppressor protein, as a stable prognostic biomarker for early-stage NSCLC [78]. BTG2 expression is consistently associated with improved survival rate across individual cohorts, with hazard ratios (HRs) ranging from 0.28 to 0.68. Beyond early-stage disease, multi-omics approaches are also important to monitor lung cancer in advanced lung cancer. For example, pretreatment levels of surfactant protein B (SP-B)–specific IgG autoantibodies and CD4^+^ T cells, identified through scRNA-seq and Olink proteomics analyses, may serve as potential biomarkers for predicting the likelihood of developing ICI-induced immune-related adverse events (irAEs) [79]. Biomarkers in predicting clinical outcome of SCLC have been identified recently. For example, RE1 silencing factor (REST), a vital transcriptive regulator corelated with neuronal differentiation, may act as a predictive biomarkers for the survival of low-NE SCLC [80]. By integrating scRNA-seq and digital special profiling (DSP), NE features and immune infiltration heterogeneity in SCLC are fully depicted in multiple dimensions. NE-low tumors are strongly related with immune infiltration including M1- and M2-like macrophages, B cells, monocytes and CD4^+^/CD8^+^ T cells, indicating better prognosis. These immune cells mostly locate in the surrounding areas of tumor nests. Within tumor nest, high ratio of tumor-infiltrating B cell may associate with low TLS formation and worse OS.

### Multi-omics analysis facilitates predicting recurrence of lung cancer

Multi-omics biomarkers play a vital role in uncovering insights into lung cancer recurrence. Predicting the subclonal nature of subsequent recurrence could significantly enhance the development of precision adjuvant therapies aim at preventing subclone-driven lung cancer relapse. An integrated multi-omics study, incorporating WES, RNA-seq, scRNA-seq, and DNA methylation profiling analyses, has shown landscapes of alteration in intratumor cells and TME in multiple dimensions and classified patients of recurrent stage I NSCLC into 4 subclusters for prediction [81]. Genomic instability such as TP53 mutation and DNA hypomethylation such as preferentially expressed antigen in melanoma (PRAME) contribute to recurrent stage I NSCLC as genetic and epigenetic biomarkers. Alterations in the TME are also pivotal in lung cancer recurrence featured with the enrichment of AT2-derived malignant cells, exhausted CD8^+^ T cells, and SPP1^+^ macrophages. Furthermore, 4 subclusters correlated with recurrent stage I NSCLC are divided into high-risk (nmf1, nmf2), moderate-risk (nmf3), and lower-risk (nmf4) to reduce recurrences and to guide precise treatment. The nmf1 subtype features enrichment of angiogenesis and the highest exhausted score. The nmf2 subtype displays a significant activation of MYC targets, G2M checkpoint pathways. And the nmf3 subtype shows high mutations of EGFR.

### AI-powered multimodal integration facilitates management of lung cancer

AI-powered multimodal integration holds promise in the field of precision oncology, enhancing treatment response prediction, or relapse forecasting in lung cancer. The integration of radiomics and genomics combine imaging features with driver gene mutations identified by WES/WGS such as EGFR, KRAS, and TP53, which helps to predict gene mutation status as well as ITH in non-invasive method and assists in dynamic risk assessment and personalized medication decisions [82]. A multimodal framework integrating medical imaging, histopathologic and genomics features predicts the response to PD-L1 blockade in NSCLC patients [83]. This multimodal model which achieves an AUC of 0.80 surpasses unimodal measures across 247 patients, including TMB (AUC = 0.61) and PD-L1 immunohistochemistry score (AUC = 0.73). LUCID, a two-stage multimodal AI framework, has been developed for predicting EGFR mutations and survival outcomes in lung cancer by incorporating CT images, patient-reported symptoms, demographic representation, and laboratory findings [84]. This model achieves an AUC of 0.881 for EGFR mutation prediction across 5,175 samples and up to 0.912 for survival prediction. Furthermore, an integrated single-cell classification model based on multi-omics biomarkers and neural networks has been constructed and validated to predict metastatic sites in LUAD patients, with an AUC of 0.87 for primary tumor and 0.87 for bone metastasis [85]. This model leverages a dynamite network biomarker approach, integrating scRNA-seq data and serum LC-MS, to identify serum-secreted proteins associated with five distinct metastatic states, including serum amyloid A1 (SAA1) as a predictive biomarker for lung cancer bone metastasis. Moreover, advancements in computational biology have facilitated the deeper investigation into tumor relapse. Notably, Generative pre-trained transformers 4 (GPT-4) has been utilized for cell-type annotation using marker genes in scRNA-seq data, enabling high-resolution tracking of cellular dynamics in recurrence [86]. These AI-driven, multimodal strategies provide powerful tools for improving clinical management of lung cancer.

## Conclusions and future prospect

Lung cancer remains one of the most formidable challenges in oncology, and early detection is critical for improving survival outcome. However, only a small proportion of patients are diagnosed at an early stage, emphasizing the urgent need for more effective screening strategies to enhance prognosis and treatment outcome [87]. Biomarkers identified by multi-omics approaches, which include genetic, epigenetic, proteomic, and metabolomic indicators, improve accuracy for early-stage detection, targeted therapy, and monitoring of lung cancer progression [88]. These breakthroughs have not only refined diagnostic precision but also paved the way for personalized treatment strategies, ultimately improving patient outcomes.

Despite the significant progress achieved through multi-omics strategies in identifying diagnostic, predictive, and prognostic biomarkers for lung cancer, several critical challenges remain. First, the complexity of multi-omics data necessitates the development of more advanced computational tools and machine learning models to facilitate effective data integration and interpretation across multiple omics layers. For instance, recent study has characterized the proteogenomic landscape of LUAD manifested as subsolid nodules, identifying three distinct molecular clusters defined by high mannose glycopeptides, paucimannose glycopeptides, and fucosylated glycans respectively [26]. The identification of these proteogenomic biomarkers relies on the integration of genomics, proteomics, phosphoproteomics, and glycoproteomics. Without robust analytical frameworks and technical equipment, the potential of multi-omics approaches may remain unrealized. Second, inter-cohort variability remains a significant barrier to the reproducibility and clinical translation of multi-omics biomarkers. Variations in sample acquisition, processing methods, storage conditions, and patient population characteristics can introduce substantial heterogeneity across dataset. These inconsistencies, in turn, compromise the generalizability and stability of multi-omics models. Furthermore, many multi-omics datasets are often characterized by high dimensionality yet limited sample sizes, which exacerbate the risk of overfitting and statistical bias. For example, multi-omics approaches combining ultra-performance liquid chromatography-tandem mass spectrometry (UPLC-MS/MS), scRNA-seq and spatial transcriptomics have identified prognostic biomarkers such as L-leucine and HMGCS1 for immunotherapy efficacy in NSCLC [89]. However, the abundance of plasma metabolites may be susceptible to biological and technical variability, which may impair the sensitivity and interpretability of such findings and limit their clinical applicability. To overcome these barriers, standardized protocols for biospecimen collection, strict quality control measures, and comprehensive documentation across studies and clinical centers are essential. Moreover, while various multi-omics biomarkers have demonstrated promise in research settings, many remain unvalidated in independent cohorts and are hindered by technical, regulatory, and cost-related barrier that limit their clinical translation. Their sensitivity and specificity have not been fully validated in clinical trials, necessitating further studies to establish their robustness and reproducibility. To bridge the gap between research and clinical application, extensive validation studies and standardized methodologies must be prioritized to ensure the successful translation of multi-omics biomarkers into clinical practice.

In conclusion, multi-omics approaches will drive significant advancements in unraveling the intricate landscape of the lung cancer ecosystem while providing deeper insights into tumor evolution and disease progression. To fully harness the potential of multi-omics in precision oncology, the development of novel omics technologies, coupled with the implementation of advanced integrative analytical frameworks, is essential. This progress will ultimately pave the way for more precise, effective, and individualized treatment strategies, significantly improving the future management and clinical outcomes of lung cancer patients.

## Data Availability

No datasets were generated or analysed during the current study.

## Abbreviations

TME: Tumor microenvironment
WGS: Whole genome sequencing
WES: Whole-exosome sequencing
scRNA-seq: Single-cell RNA sequencing
KACs: KRT8^+^ alveolar intermediate cells
AT2: Alveolar type II
LUAD: Lung adenocarcinoma
LC-MS/MS: Liquid chromatography-tandem mass spectrometry
TIM-3: T-cell immunoglobulin and mucin-domain containing-3
TMB: Tumor mutational burden
mCAFs: Matrix-associated cancer-associated fibroblasts
KRT8^+^ AT2 cell: Keratin 8-positive alveolar type II cell
CLDN2^+^ AT2 cell: Claudin-2-positive alveolar type II cell
COL6A3^+^ fibroblast: Collagen type VI alpha 3 chain-positive fibroblast
AIS: Adenocarcinoma in situ
PCSK9: Proprotein convertase subtilisin/kexin type 9
IAC: Invasive adenocarcinoma
ITH: Intratumoral heterogeneity
NSCLC: Non-small cell lung cancer
AAH: Atypical adenomatous hyperplasia
EC: Endothelial cell
NK cell: Natural killer cell
THBS2^+^ CAF: Thrombospondin-2-positive cancer-associated fibroblast
LSCC: Lung squamous cell carcinomas
TLS: Tertiary lymphoid structures
GC: Germinal center
Tfh: T follicular helper cell
HLA-II: Human leukocyte antigen class II
ECM: Extracellular matrix
LUSC: lung squamous cell carcinoma
TAM: Tumor-associated macrophage
ABCA1: ATP-binding cassette sub-family A member 1
LDLR: Low-density lipoprotein receptor
CIN^high^: Chromosomal instability high
LC-BrM: Lung cancer brain metastasis
OXPHOS: Oxidative phosphorylation
SCNA: Somatic copy number alteration
LRG1: Leucine-rich alpha-2-glycoprotein 1
cfDNA: Cell-free DNA
ctDNA: Circulating tumor DNA
SCLC: Small cell lung cancer
NE: Neuroendocrine
RB1: Retinoblastoma 1
ASCL1: Achaete-scute family bHLH transcription factor 1
NEUROD1: Neuronal differentiation 1
IFNγ: Interferon-γ
DLL3: NOTCH ligand delta-like protein 3
RTK: Receptor tyrosine kinases
IL-33: Interleukin-33
ICI: Immune checkpoint inhibitor
AUC: Area under the curve
mdMSP: Multiplex digital methylation-specific PCR
AI: Artificial intelligence
CEA: Carcinoembryonic antigen
NPLDI-MS: Nanoparticle-assisted laser desorption/ionization mass spectrometry
PD-L1: Programmed death-ligand 1
ICB: Immune checkpoint blockade
OS: Overall survival
ACh: Acetylcholine
TKI: Tyrosine kinase inhibitors
M3R: Ach muscarinic receptor 3
BTG2: B-cell translocation gene 2
HR: Hazard ratio
irAEs: Immune-related adverse events
SP-B: Surfactant protein B
REST: RE1 silencing factor
DSP: Digital special profiling
SAA1: Serum amyloid A1
GPT-4: Generative pre-trained transformers 4
UPLC-MS/MS: Ultra-performance liquid chromatography-tandem mass spectrometry
MIA: Minimally invasive adenocarcinoma
KRAS: Kirsten rat sarcoma viral oncogene
EGFR: Epidermal growth factor receptor
TP53: Tumor protein p53
PRAME: Preferentially expressed antigen in melanoma
CDKN2A: Cyclin dependent kinase inhibitor 2 A
